# sccomp: Robust differential composition and variability analysis for single-cell data

**DOI:** 10.1073/pnas.2203828120

**Published:** 2023-08-07

**Authors:** Stefano Mangiola, Alexandra J. Roth-Schulze, Marie Trussart, Enrique Zozaya-Valdés, Mengyao Ma, Zijie Gao, Alan F. Rubin, Terence P. Speed, Heejung Shim, Anthony T. Papenfuss

**Affiliations:** ^a^Bioinformatics Division, The Walter and Eliza Hall Institute of Medical Research, Parkville, VIC 3052, Australia; ^b^Department of Medical Biology, University of Melbourne, Parkville, VIC 3052, Australia; ^c^Melbourne Integrative Genomics, University of Melbourne, Parkville, VIC 3052, Australia; ^d^School of Mathematics and Statistics, University of Melbourne, Parkville, VIC 3052, Australia

**Keywords:** single-cell, cell-type proportion, compositional, variability, microbiome

## Abstract

Cellular omics such as single-cell genomics, proteomics, and microbiomics allow the characterization of tissue and microbial community composition, which can be compared between conditions to identify biological drivers. This strategy has been critical to revealing markers of disease progression, such as cancer and pathogen infection. A dedicated statistical method for differential variability analysis is lacking for cellular omics data, and existing methods for differential composition analysis do not model some compositional data properties, suggesting there is room to improve model performance. Here, we introduce sccomp, a method for differential composition and variability analyses that jointly models data count distribution, compositionality, group-specific variability, and proportion mean–variability association, being aware of outliers. sccomp provides a comprehensive analysis framework that offers realistic data simulation and cross-study knowledge transfer. Here, we demonstrate that mean–variability association is ubiquitous across technologies, highlighting the inadequacy of the very popular Dirichlet-multinomial distribution. We show that sccomp accurately fits experimental data, significantly improving performance over state-of-the-art algorithms. Using sccomp, we identified differential constraints and composition in the microenvironment of primary breast cancer.

## Significance

Determining changes in the composition of cell populations is made possible by technologies like single-cell transcriptomics, CyTOF, and microbiome sequencing. However, existing methods for differential abundance do not model some compositional count data properties, and dedicated models do not yet handle cell-group-specific differential variability. A suitable statistical method would enable analyses to identify component-specific loss of homeostasis. Developing a constrained Beta-binomial distribution, we have implemented a statistical model, sccomp, that enables differential variability analysis for compositional data, improved differential abundance analyses with cross-sample information borrowing, outlier identification and exclusion, realistic data simulation, and cross-study knowledge transfer.

Composition analysis models the proportion of cell types, taxa, or other entities in a population. Tissue composition analysis enabled seminal discoveries in cancer research (1–6), epidemiology, metabolic disease (7) and skin physiology (8). Single-cell transcriptomics (9) and high-throughput flow cytometry (CyTOF) (10) enable the characterization of cell groups by measuring the abundance of thousands of transcripts and tens of proteins at the single-cell level. The 16S rRNA and whole-microbiome DNA sequencing characterize bacterial taxonomic groups (8) by probing their genetics. The relative abundance of groups of cells or microorganisms can be compared between biological or clinical conditions to identify cellular or taxonomic drivers.

Highlighting the importance and the challenges of analyzing compositional data using cellular omics, several statistical approaches have been developed. Compositional data possess several key statistical properties that these methods model in various combinations (Table 1). Well-known properties are: i) data are observed as counts; ii) group proportions sum to one and are negatively correlated (which we term compositionality); and iii) proportion variability is group-specific. Methods such as scDC (11), propeller, and diffcyt (12) use linear regression, based on log or arcsin-square-root-transformed proportions, to model data compositionality (ii) and handle group-specific variability (iii) but do not model the data count distribution. Modeling single-cell compositional data as counts is important as small datasets and rare cell types are characterized by a high noise-to-signal ratio, and modeling counts enables the down-weighting of small cell-group proportions compared to larger ones. Log-count-based methods such as MixMC (13), Bach et al. (14), and ANCOM-BC (15) model group-specific variability (iii) but do not model counts or data compositionality. Binomial-based methods such as those used in Pal et al. (16), and corncob (17) model counts (i) and cell-group-specific variability (iii) but do not model the compositionality (ii). Multinomial-based methods such as ALDEx2 (18), dmbvs (19), and scCODA (20) model count data (i) and compositional properties (ii) but assume the same variability for all groups.

**Table 1.** Properties of compositional methods for single-cell data

Method properties									
I. Data are modeled as counts									
II. Group proportions are modeled as compositional									
III. The proportion variability is modeled as cell-type specific									
IV. Information sharing across cell-types, mean–variability association									
V. Outlier detection or robustness									
VI. Differential variability analysis									
Methods	Year	Model	I	II	III	IV	V	VI	Reference
sccomp	2023	Sum-constrained Beta-binomial	●	●	●	●	●	●	Mangiola et al.
scCODA	2021	Dirichlet-multinomial	●	●					Buttner et al. (20)
quasi-binom.	2021	Quasi-binomial	●		●				Pal et al. (16)
rlm	2021	Robust-log-linear		●			●		Bach et al. (14)
propeller	2021	Logit-linear + limma		●	●	●			Phipson et al. (21)
ANCOM-BC	2020	Log-linear		●	●				Lin et al. (15)
corncob	2020	Beta-binomial	●		●				Martin et al. (17)
scDC	2019	Log-linear		●	●				Cao et al. (11)
dmbvs	2017	Dirichlet-multinomial	●	●					Wadsworth et al. (19)
MixMC	2016	Zero-inflated Log-linear		●	●				Cao et al. (13)
ALDEx2	2014	Dirichlet-multinomial	●	●					Fernandes et al. (18)

Other important data properties, such as the proportion mean–variability association (iv) and the presence of outliers (v), have remained largely uncharacterized. A formal description of the mean–variability association across cellular omics technologies and incorporation into a statistical model would allow differential variability analysis and imply the inadequacy of single variability distributions, such as the Dirichlet-multinomial, widely applied for count-based compositional analyses. Characterizing the impact of outliers would enable the development of robust methods for cellular omics data.

For cellular omics data, dedicated models have not handled differential variability analysis. Differential variability analysis is an avenue for novel discoveries through single-cell transcriptomics, such as for T cell response in cancer (22). The increase in the variability of tissue composition and microbial communities is a well-known indicator of loss of homeostasis and disease. A suitable statistical method would enable to identify component-specific loss of homeostasis.

Here, we introduce sccomp, a generalized method for differential composition and variability analyses based on sum-constrained independent Beta-binomial distributions. This method takes into account the five statistical properties of cellular omics–based compositional data. Furthermore, sccomp can simulate realistic data with the properties of any experimental dataset. The simulated data can be used to assess the adequacy of the fitted model and for benchmarking purposes. Our model can incorporate knowledge from previously modeled datasets as prior information to improve estimates for small query datasets.

Applying sccomp to 18 datasets, we characterize the mean–variability relationship of compositional data across cellular omics technologies, including single-cell RNA sequencing, CyTOF, and microbiome profiling. Our findings suggest that the Dirichlet-multinomial distribution is inadequate to model the differential composition of those omic technologies and that incorporating the mean–variability relationship is required for differential variability analysis. Our results also show the ubiquitous presence of outlier observations in all datasets. Using realistic simulations, we show that sccomp significantly improves performance compared to other methods. Our method uncovered differential microenvironmental constraints of breast cancer subtypes and cell-type-specific differences involving lymphoid and myeloid cell populations. Uniquely, the sum-constrained Beta-binomial distribution allows the modeling of the compositional properties of data with mean–variability association while allowing for outlier exclusion; we anticipate its adoption in other scientific fields.

## Results

### Overview of sccomp.

**Fig. 1.**
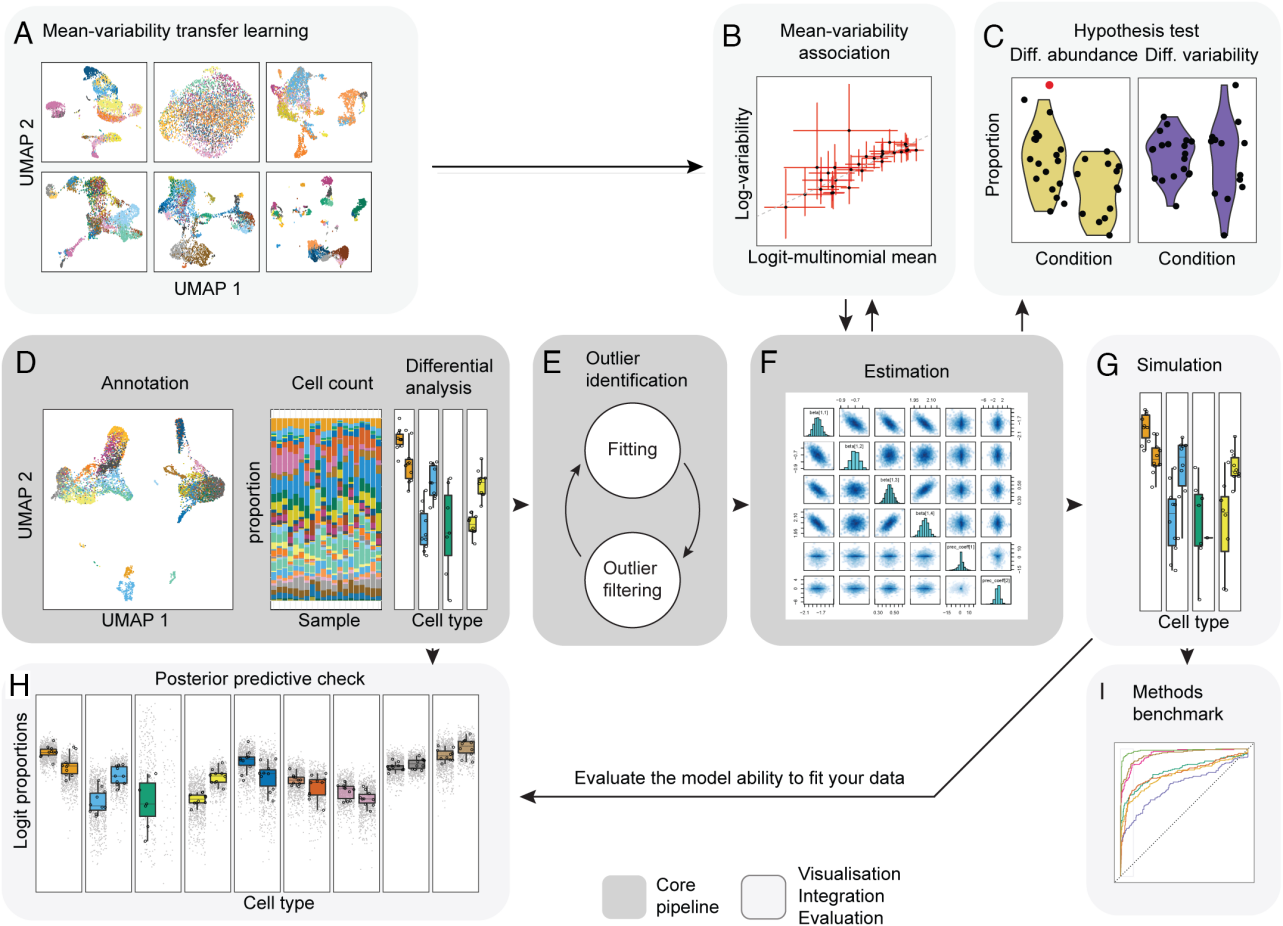
sccomp core algorithm, data integration, and visualization. (*A*) Integrating existing single-cell compositional studies gives prior information on the proportion mean–variability association (*Cross-dataset learning transfer* in *Methods*). (*B*) Representation of the association between proportion means and variability (*Statistical model* in *Methods*). (*C*) An example of the difference in cell-group abundance (left-hand side) and variability (right-hand side) that sccomp can estimate (*Differential variability analysis* in *Methods*). (*D*) Representation of the process from cell clustering and counting that is the input for the differential composition analysis (*User interface* in *Methods*). (*E*) Schematic of the iterative process of outlier identification and exclusion (*Iterative outlier detection* in *Methods*). (*F*) Illustration of the posterior probability distribution of regression coefficients from the model fitting (Hypothesis testing in *Methods*). (*G*) Data simulation from the fitted model. (*H*) Posterior predictive check simulates data under the fitted model and then compares these to the observed data (25) (Posterior predictive check, *Methods*). This check allows users to evaluate the ability of the model to fit a specific input dataset. (*I*) Representation of benchmarking with realistic data that sccomp allows in a user-friendly way.

To model the count and proportional properties of single-cell compositional data while allowing for cell-group-specific variability and outlier identification (Table 1), we developed sccomp. This method underlies a Bayesian model based on sum-constrained independent Beta-binomial distributions. sccomp can simultaneously estimate differences in composition and variability (Fig. 1*C*) from complex experimental designs, including discrete and continuous covariates. The estimation is done through Hamiltonian Monte Carlo via the Bayesian inference framework Stan (23). Hypothesis testing is performed by calculating the posterior probability of the composition and variability effects being larger than a specified fold-change threshold (24). Estimation is made more stable with an adaptive shrinkage in the form of a data-learned prior distribution defining the association between proportion means and variabilities (Fig. 1*B* and *Methods*). Optionally, sccomp identifies outliers probabilistically through iterative fitting (Fig. 1*E* and *Methods*), which are excluded from later fits (Fig. 1*F*).

Additionally, sccomp allows the incorporation of the mean–variability association from other datasets (Fig. 1*A*). This prior knowledge is beneficial when only a few groups or samples are present, posing a challenge in estimating this association. After learning the data proprieties through model fitting, sccomp can simulate realistic datasets (Fig. 1*G*). Simulated data can help identify potential failings of the model (i.e., through posterior predictive check; Fig. 1*H*) and enables benchmarking based on more realistic simulations (Fig. 1*I*). The execution time of sccomp (version 1.3.5) ranges from 7 s for tiny datasets (four samples, five cell groups) without outlier detection to 120 s for larger datasets (20 samples, 20 cell types) with outlier detection.

### sccomp Improves the Performance of Differential Compositional Analyses.

**Fig. 2.**
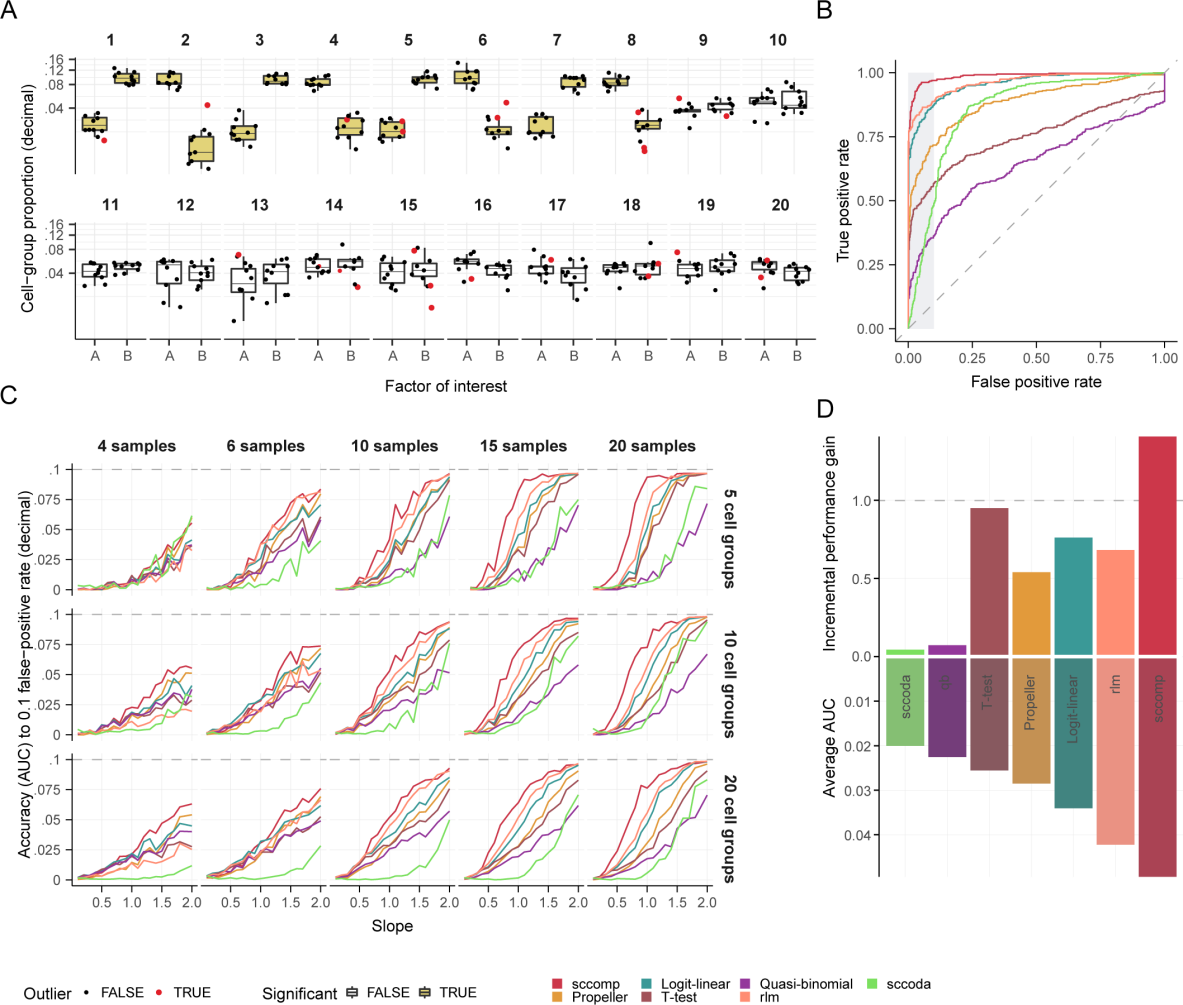
sccomp outperforms state-of-the-art methods for realistic data simulations (including outliers) based on a logit-linear-multinomial model of the COVID-19 dataset EGAS00001004481 (4) (see *Methods* section Benchmark). (*A*) Example of a simulation with the following settings: regression slope of 1.5, 20 samples (10 per condition), 20 groups, 1,000 total cells per sample, with 8 groups (40%) being differentially abundant and 12 having no differences. The yellow groups are differentially abundant. (*B*) The ROC curve for the simulation in panel *A*, measuring the ability of the methods to identify groups as different or not based on the ground truth, as the threshold is varied. The gray area represents the false positive threshold used to calculate the AUC, which indicates the relative performance of each method. (*C*) The benchmarking across a range of slopes, number of samples and groups. Each performance measure represents an average of 50 areas under the curve (up to the 0.1 false-positive rate) for 50 simulations with the same parameters. (*D*) Incremental performance gain across all simulation conditions (*Methods*) of sccomp compared to other methods. A onefold gain represents a linear incremental gain along the methods rank. Methods are ordered by their average performance across simulation conditions (bottom facet).

We evaluated whether our modeling strategy benefits the estimation of differences in single-cell compositional data. We compared the performance of sccomp with publicly available methods for differential composition analysis (Table 1), performing a benchmarking on realistic simulated data based on the noise and outlier characteristics of the COVID-19 dataset (4) (Fig. 2*A* and *SI Appendix, Methods*). The simulation was based on a logit-linear-multinomial distribution to ensure fairness across methods. We built a receiving-operator characteristic (ROC) curve for every run and evaluated the performance using the area under the curve (AUC, up to 0.1 false-positive rates; Fig. 2*B*).

The method sccomp outperformed other methods (Fig. 2*C*). The performance gap incrementally improved as the simulated data’s effects increased, reaching a plateau at an average AUC of 0.1. The performance gap further widened in sum-constrained Beta-binomial simulations (1.4 and 1.9-fold improvement; *SI Appendix*, Fig. S1). The method sccomp had the highest performance gain among other methods (Fig. 2*D*), being the only method with a greater-than-linear gain in the method performance rank. Rlm and logit-linear were the second- and third-best performers (0.64 and 0.75 incremental gain, respectively). Across simulations, the number of groups had little impact on performance. In the benchmark based on outlier-free data simulation, sccomp’s performance was still superior, with smaller incremental improvements from the other methods (*SI Appendix*, Fig. S2).

Our method can improve estimates by transferring information from publicly available datasets (see *Cross-dataset learning transfer* subsection). To test the effectiveness of this technique to regularize estimates in low-data settings, we compared the use of uninformative or informative hyperpriors. Our results show improvements in performance for datasets with low sample sizes (n = 2 to 4) and small differences between conditions (e.g., treated versus untreated; *SI Appendix*, Fig. S3). The performance improvement is not significantly affected by the choice of reference dataset as long as it is generated from the same data modality (e.g., 10x single-cell RNA sequencing). For extremely low sample size datasets and small effects, both the ideal and alternative single-cell RNA reference confer an equivalent improvement in performance. The performance benchmark with an extremely misleading and confident (i.e., small SD) hyperprior negatively affects the performance for low-sample and group-size datasets (*SI Appendix*, Fig. S3) while it does not have a large effect from a sample size of six.

### sccomp Identifies Differential Constraints across Cancer Subtypes in the Breast Microenvironments.

**Fig. 3.**
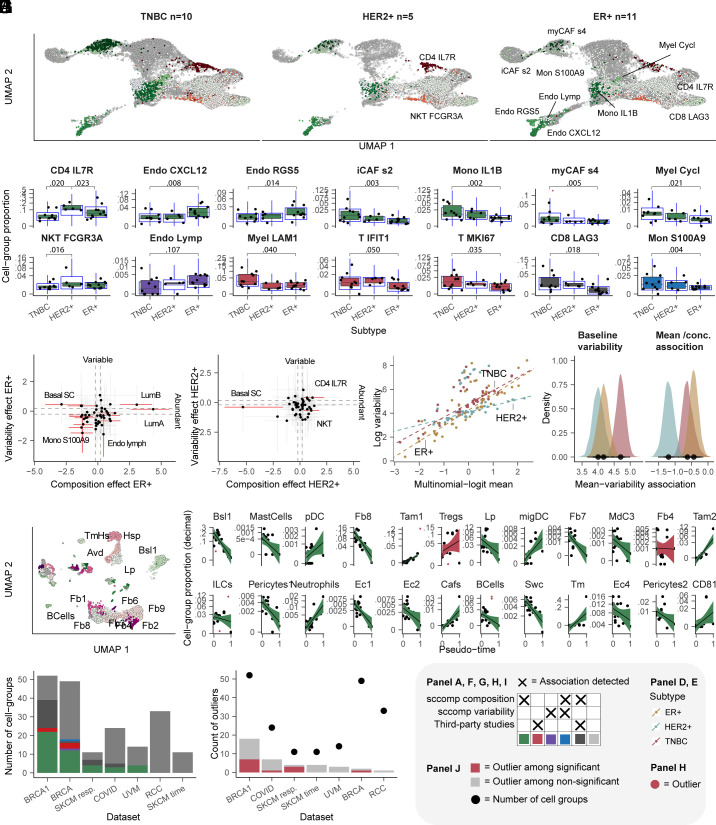
sccomp reveals novel results from public data from Wu et al. (3) and five single-cell RNA sequencing datasets (1, 3–5, 14). (*A*) UMAP projection of cells for three breast cancer conditions (subtypes). Cells are shaded according to the type of finding (e.g., green shade for differential composition associations). As triple-negative (TNBC) was compared with the other two conditions, only the cell groups with new findings were labeled for HER2+ and ER+ facets. (*B*) Proportion distributions of the cell types with novel results (both positive and negative). The blue box plots represent the posterior predictive check. (*C*) Correlation of the estimated difference in composition (*x* axis) and variability (*y* axis) for the triple-negative versus ER+ comparison. Error bars are the 95% credible interval. Red error bars represent significant associations. Gray dashed lines represent the minimum difference threshold of 0.2. Significant associations for cancer populations are shown in *SI Appendix*, *Methods*. (*D*) Correlation of the estimated difference in composition and variability for the TNBC versus HER2+ comparison. (*E*) Mean–variability associations (log scale) for the three cancer conditions (see *SI Methods* subsection *Reanalysis of single-cell RNA sequencing datasets*). Each dot represents a cell group. The dashed lines are the sccomp estimate of such an association. (*F*) Posterior distributions of the intercept and slope parameters for the three conditions, shown in panel *E*. (*G*) UMAP projection of cells for the Bach et al. (14) dataset. Cells are shaded according to the type of finding. Only cell groups part of novel findings are labeled as text. (*H*) Proportion distributions of the cell types with the novel (green, red, purple, blue) and non-novel (dark and light gray) results. (*I*) Count of cell groups for each dataset and the number of consistent, novel, and rejected associations. The datasets are ordered by the number of novel results. (*J*) Number of outliers for each dataset. Red represents outliers identified for differentially abundant cell groups. Dots represent the number of cell groups per dataset. The datasets are ordered by the number of outliers identified.

We used sccomp to analyze the microenvironment of primary breast cancer from data first described by Wu et al. (3) (*SI Appendix*, *Methods*). This study analyzed 26 breast cancer primary tumor tissues across three subtypes (TNBC, ER+, HER2+) and identified 49 cell phenotypes. We analyzed the difference in composition and variability of the triple-negative subtype (TNBC) compared to ER+ and HER2+ identifying a diverse landscape of compositional and variability changes across subtypes (Fig. 3*A*). The main feature is the depletion of cytotoxic CD8 IFN-γ in TNBC, compared to HER2+ and ER+ (Fig. 3*B*). Compared with TNBC, the HER2+ microenvironment is enriched in several other lymphocytic populations, including CD4 follicular helper (CD4 fh in Fig. 3*B*), CD4 CCR7+, CD4 IL7R+, T regulatory (T-reg), natural killer (NK AREG), and NKT (Fig. 3*B*). ER+ tumors are characterized by changes in the stromal compartment, with enrichment of endothelial cells (endo ACKR1, CXCL12, RGS5) and depletion of cancer-associated fibroblasts (iCAFs2 and myCAFs4), inflammatory monocytes (Mon S100A9), and B naive cells, compared with TNBC (Fig. 3*B*). The differences identified by Wu et al. in the immune/stromal compartments using a *t* test (3) were not labeled significant by sccomp; however, the estimated signs agree. The estimated enrichment of the cancer cell phenotypes (Basal, luminal, HER2+) for the respective clinical subtypes is consistent with Wu et al. (3) (*SI Appendix*, Fig. S4*A*).

Most importantly, sccomp allowed the investigation of latent microenvironmental constraints across breast cancer subtypes (see *SI Methods* subsection *Reanalysis of single-cell RNA sequencing datasets*). Hidden from the analyses of single groups (Fig. 3 *A* and *B*), the overall proportional variability within groups (intercept of the mean–variability regression line; Fig. 3 *E* and *F*) for TNBC is significantly higher than for the other two subtypes. This trend indicates an overall higher microenvironmental heterogeneity across patients. Also, while ER+ and TNBC share a similar mean–variability association (slope of −1.3 and −1.1; Fig. 3 *A* and *B*), HER2+ shows a distinct cohort-level heterogeneity profile. A markedly smaller slope indicates a more similar relative variability across cell types and potentially distinct microenvironmental processes acting for this condition.

### sccomp Leads to Novel Discoveries from Public Datasets.

To further assess the ability of sccomp to generate novel results, we expanded our analysis on a time-resolved BRCA1 model of tumorigenesis (E-MTAB-10043; 14) where the samples were assigned to a pseudotime continuous coordinate as defined in the *SI Appendix*, *Methods*. This study used a robust log-linear model and a robust F test to estimate 17 significant differences along the tumor developmental timeline, including fibroblast, dendritic, monocyte, and T cells. We confirmed most of those associations and identified 15 new associations, such as tumor-associated fibroblasts (Fb7, Fb8) and macrophages (Tam1, Tam2), neutrophils, and mig dendritic cells (migDC). Five associations proposed by the study were labeled nonsignificant by sccomp (Fig. 3*H*), two including outliers.

To assess the usefulness of sccomp more broadly, we analyzed four other single-cell RNA sequencing public datasets (*SI Appendix*, Table S1). This analysis generated novel results, including differential composition and variability in all datasets (Fig. 3*I* and *SI Appendix*, Fig. S4*B*). sccomp identified outliers in all datasets, with 19% of cell groups containing one or more (Fig. 3*J*). In addition, 20% of the outlier-positive cell groups, which previous analyses did not label as significant, were labeled as significant by sccomp after excluding outliers. The comparison between the original and sccomp analyses revealed that 15% of the disagreed calls included one or more outliers.

### Proportion Means and Variabilities Are Log-Linearly Correlated in Cell-Omic Data.

**Fig. 4.**
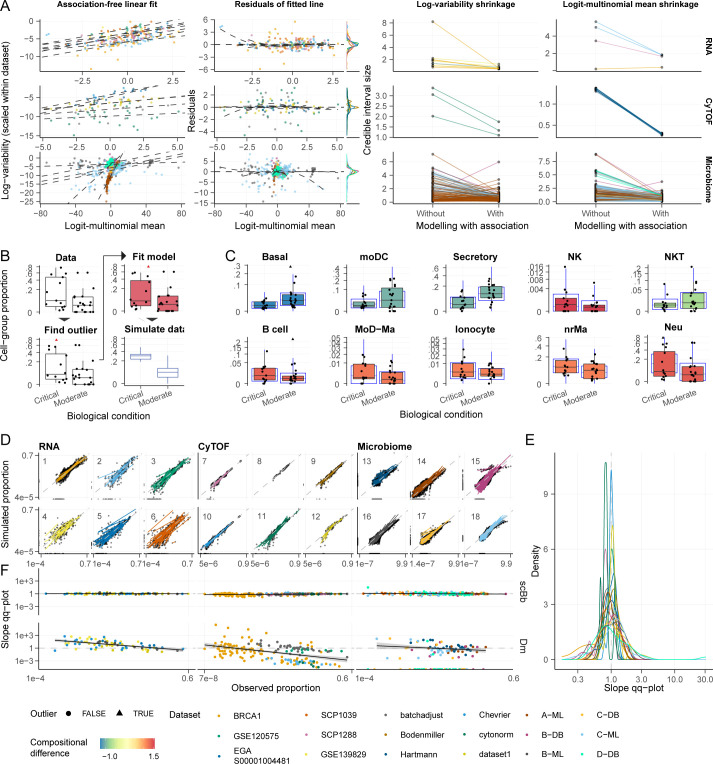
Sum-constrained Beta-binomials modelling mean–variability associations are adequate for experimental data from 6 studies (*SI Appendix*, Table S1). (*A*) Study of the correlation between the proportion mean and variability (see *Methods* subsection *Study of mean–variability association*). The left facets refer to mean and variability estimates association without constraints on their relationship. The dotted line is the fit of robust linear modeling [rlm (26)]. The middle facets plot the rlm residuals versus fitted values with a lowess smoother superimposed. The facets on the right show a decrease in the size of the 95% credible intervals for all datasets. Only changes larger than 0.5 are shown (increase or decrease). (*B*) The four main steps of the sccomp algorithm (see *Methods* section Study of model adequacy to experimental data). (*C*) Example of the posterior-predictive check with the simulated data over the observed data [colorful boxplot, COVID-19 dataset EGAS00001004481 (4); blue boxplots]. The subset of cell groups showing a larger effect is visualized. The color code expressed the magnitude of the difference estimated by sccomp across biological conditions, critical and moderate. (*D*) Scatter plot of the observed versus simulated cell-group proportions for 6 datasets. Datasets are labeled by their numeric IDs (*SI Appendix*, Table S1). Each point corresponds to the proportion of a sample-cell group combination, and each line corresponds to a cell group. The slopes of fitted lines describe the match between observed and generated data for one group (paired by their ranks), which is expected to be 1 when the two distributions are the same. The dashed gray line represents a perfect linear match. (*E*) The distribution of slopes of the scatter plots (panel *D*). (*F*) Association between the slopes of the scatter plots of the observed (*y* axis) and each group’s estimated (*x* axis) proportion abundance. The sum-constrained Beta-binomial (scBb) and Dirichlet-multinomial (Dm) are compared. If the data simulated from the posterior predictive distribution are similar to the observed data, we expect a straight horizontal line with intercept 1.

To evaluate the association between proportion mean and variability, we analyzed 18 datasets across single-cell RNA sequencing, CyTOF, and microbiome profiling technologies (*SI Appendix*, Table S1). We first used the sum-constrained Beta-binomial model with no built-in mean–variability association (see *Methods* for notation). We then examined the correlation of the independently estimated means (logit-multinomial link) and variabilities (log link). We consistently observed positive linear homoscedastic association for all three technologies (Fig. 4 *A*, *Left* and *SI Appendix*, Fig. S5, dotted line and residuals; *SI Appendix*). We then compared these mean and variability estimates to the ones produced with the sum-constrained Beta-binomial model with built-in mean–variability association. This comparison shows that the hierarchical modeling of the mean–variability association confers a significant shrinkage of the variability estimates up to four-fold (Fig. 4 *A*, *Right* and *SI Appendix*, Fig. S5*D*).

For single-cell RNA sequencing data, modeling this association had a shrinkage effect on the variability estimates (and means to a lesser extent), something obvious for the BRCA1 dataset for cell types with low abundance (e.g., tumor-associated macrophages, Tam1, *SI Appendix*, Fig. S5). For CyTOF data, the shrinkage effect is evident in the Bodenmiller and CytoNorm datasets. Similarly, the most significant impact can be seen for rare cell types. Microbiome data are characterized by higher uncertainty and greater spread around the regression line (before shrinkage). The shrinkage effect is more dramatic for microbiome data than other data types, especially for the means.

The estimated slope of the linear relationship is relatively consistent across technologies. The average slopes across datasets are 0.84, 0.47, and 0.55 for single-cell RNA, CyTOF, and microbiome (SDs 0.10, 0.22, and 0.26), respectively. Their intercepts are more variable, with the average means being −4.32, −7.19, and −5.66 and the SDs being 1.05, 1.86, and 5.66, respectively. Some single-cell RNA sequencing datasets show a bimodal association, where the second mode represents high-variability groups (dataset BRCA; *SI Appendix*, Fig. S5*A*). This pattern is observable in the resulting bimodal residual distribution (Fig. 4 *A*, *Middle* and second rows of *SI Appendix*, Fig. S5 *A*–*C*). To accommodate this pattern, our model allows a Gaussian mixture distribution that accurately fits both modes (*SI Appendix*, Fig. S5*A*, third row; dashed lines; *SI Appendix*).

### The Sum-Constrained Beta-Binomial Adequately Models Experimental Data across Technologies.

Our method can simulate realistic data based on the learned characteristics of experimental datasets (Fig. 4*B*). This simulation is achieved by estimating the posterior distribution from a given dataset and producing data from the posterior predictive distribution. The posterior predictive check (27, 28) helps assess the model’s descriptive adequacy for specific datasets and study designs. For example, overlaying simulated to experimental data, we show the descriptive adequacy of sccomp for the COVID-19 dataset EGAS00001004481 (4) (*SI Appendix*, Table S1) replicating interquartile ranges (Fig. 4*C*) and the absence of noticeable pathologies. To provide a more quantitative assessment, we regressed the observed and simulated data for each cell type of 18 publicly available datasets (1–5, 14, 29–32) across three cellular omics technologies (Fig. 4*D*). The fitted lines are tightly centered around the 45° reference line for all datasets (Fig. 4*E*). This evidence suggests that the proportion mean and variability relationship is descriptively adequate for and representative of experimental data across technologies. This trend is particularly significant considering that performing posterior predictive checks on small sample-size datasets suffers from noise.

### The Sum-Constrained Beta-Binomial Is a More Accurate Model for Within-Group Variability Compared to the Dirichlet-Multinomial.

Considering the existence of a mean–variability association, we assessed the ability of our model to capture the variability of small and large groups adequately. We analyzed the relationship between fitted slopes between observed and simulated proportions and the baseline abundance (estimated intercept) across 18 datasets (*SI Appendix*, Table S1). We compared our model with the Dirichlet-multinomial model, a de facto standard for count-based compositional analyses (19, 20, 33–36).

Using our model, we saw no bias in the fitted slopes of observed-simulated data across group abundance. These results indicate that our model does not significantly underestimate or overestimate the data variability for any group, regardless of their relative abundance and the data source (Fig. 4 *F*, *Top*). On the contrary, the Dirichlet-multinomial underestimates the variability for low-abundant cell groups and overestimates the variability for abundant cell groups (Fig. 4 *F*, *Bottom*) for single-cell transcriptomic, CyTOF, and microbiome data. For single-cell RNA sequencing data, the variability of small groups is consistently overestimated because of the low data support (small sample size and low cell count). In contrast, for CyTOF and microbiome, where more data are available, the consistent overestimation for small groups is mirrored by an underestimation for large ones.

### The Sum-Constrained Beta-Binomial Distribution Models Compositionality while Allowing for Group-Specific Variability.

The sum-constrained Beta binomial distribution is related to the Dirichlet-multinomial in that both have a sum-to-one constraint on the unobserved proportions. However, the former distribution is more flexible than the Dirichlet-multinomial because it can also model cell-group-specific variabilities. To test the ability of our model to capture the negative correlation between cell-group proportions, we fitted datasets simulated from the Dirichlet-multinomial distribution, and compared them with the posterior predictive distribution from the sum-constrained Beta-binomial distribution model.

**Fig. 5.**
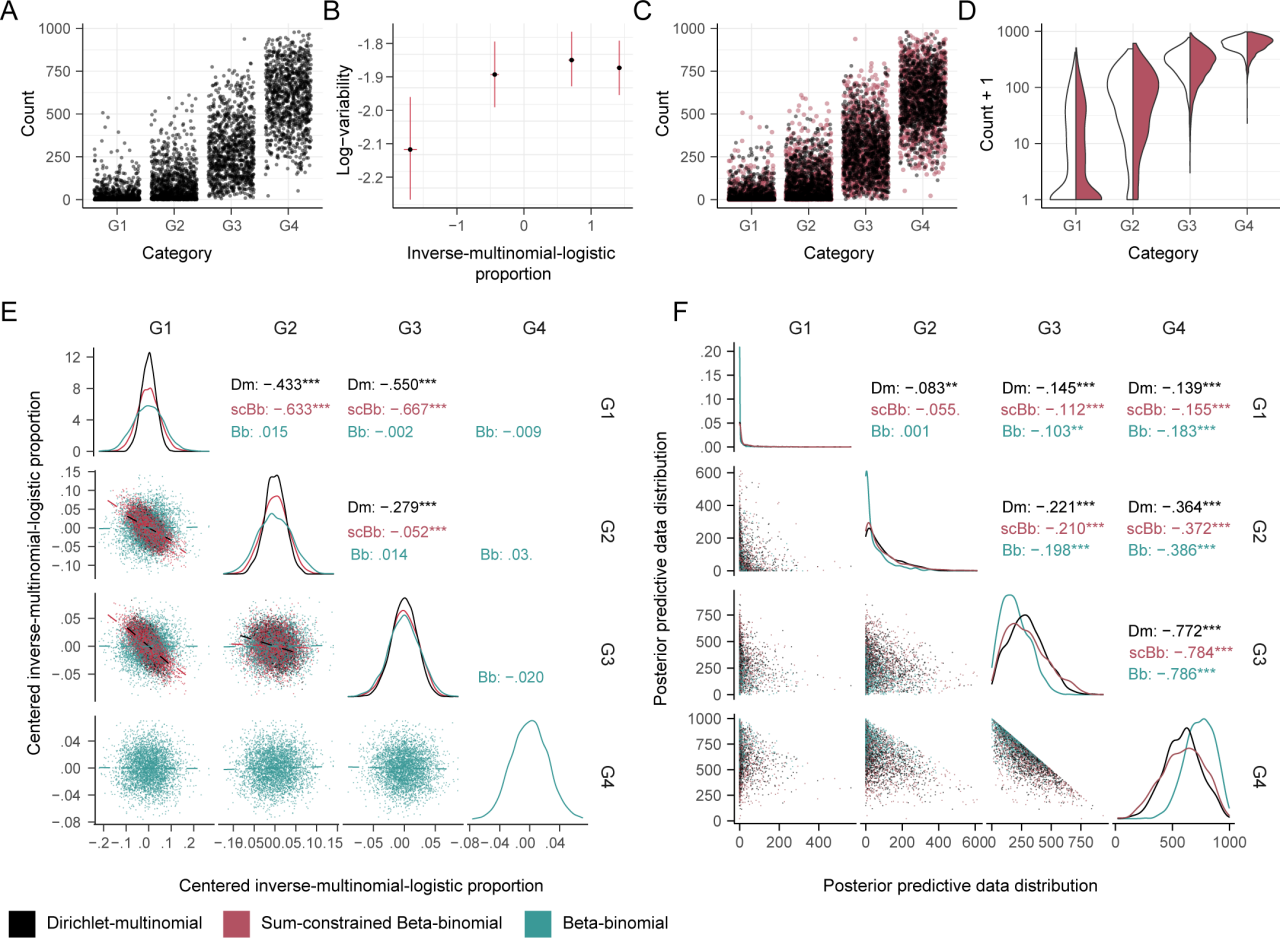
The sum-constrained Beta-binomial models the compositionality of four groups (G1, G2, G3, and G4) proportions while allowing group-specific variability. (*A*) Distribution of data simulated from a four-group Dirichlet-multinomial. (*B*) Estimated mean and variability parameters from the sum-constrained Beta-binomial. The error bars represent the 95% credible intervals. (*C*) Distribution of observed data (from *A*, black) overlaid to simulated data from the fitted model (red). (*D*) Matching densities of the observed (white) and generated data (red) for the four groups. (*E*) Draws from the posterior distribution of the scaled means (log-scale). The models used for estimation were Dirichlet-multinomial (Dm), (unconstrained), Beta-binomial (Bb), and sum-constrained Beta-binomial (scBb). The estimate for G4 is missing from Dirichlet-multinomial and sum-constrained Beta-binomial because it is not part of the parameter space but rather calculated as the negative sum of G1-3. The correlation is shown for each model. The stars indicate the correlation significance test [***=<0.001, calculated with GGally (37)]. (*F*) Overlap between the observed and generated data between each group across models.

Data were generated by a four-group Dirichlet-multinomial (with parameters 0.2, 0.6, 2.0, 4.0; Fig. 5 *A* and *B*), and the sccomp single-mean model was fitted to these data. The overlay of the posterior predictive distribution on the simulated data shows that the densities match (red data points, Fig. 5 *C* and *D*). We tested whether our model could capture the dependence structure across the proportion means, typical of compositional data, analyzing the correlation among estimated means using pairs plots. We also compared the estimated means for a Dirichlet-multinomial (as a baseline) and an unconstrained (independent) Beta-binomial model. The estimated means of our model show a negative correlation structure like the Dirichlet-multinomial model (Fig. 5*E*). This correlation is strong for groups one and two (G1 and G2) and, to a lesser extent, for group three. On the contrary, the unconstrained Beta-binomial does not reproduce this dependence. This lack of dependence results in a higher uncertainty around the estimates, especially for low-abundance groups G1 and G2.

The differences between sum-constrained and unconstrained Beta-binomial models are reflected in the ability to simulate representative data to the Dirichlet-multinomial (Fig. 5*F*). The marginal distributions of the predictive posterior and the weak dependence structure of the simulated data across the four groups, characteristic of the Dirichlet-multinomial, are accurately reproduced by the sum-constrained Beta-binomial. On the contrary, the unconstrained Beta-binomial generates visibly distinct data densities compared to the Dirichlet-multinomial.

## Discussion

As the adoption of single-cell technologies increases, the development of tailored, flexible, and robust compositional analysis methods is essential to identify changes in tissue composition between conditions. sccomp is a method for differential analysis of count-based compositional data. It is based on sum-constrained independent Beta-binomial distributions that share compositional characteristics with the Dirichlet-multinomial but allow group-specific variability and exclusion of outlier observation from the fit. Our model shares some features with the generalized Dirichlet-multinomial (38). However, it allows for missing observations and suits outlier exclusion.

The present study describes the proportion mean–variability association for cellular omics compositional data. This association is linear between logit-multinomial means and log variabilities, and can be bimodal for some single-cell RNA sequencing datasets. We tested such associations across 18 single-cell RNA sequencing, CyTOF, and microbiome datasets. Our results challenge the use of the Dirichlet-multinomial distribution, a standard in count-based compositional analysis, and the use of unconstrained, independent distributions. We showed that cellular omics compositional data (e.g., EGAS00001004481) with N groups can be modeled with no more than N+1 degrees of freedom (N-1 for the means and 2 for the variability). This finding implies that such unconstrained models tend to be heavily overparameterized (using 2N degrees of freedom). Our description of mean–variability association also has implications for differential variability testing. Ignoring the mean–variability association would result in biased estimates of the differential variability necessarily associated with the differential composition estimates. Defining the correlation line in log space allowed us to disentangle differential composition and variability straightforwardly and provide a meaningful estimate of how cell/taxonomic proportion variability varies across samples.

While the impact of outlier observations has been approached for metagenomic data (39), our study supports that single-cell compositional data are also outlier-rich. Our outlier identification probabilistic approach overcomes the challenges of using residuals to identify outliers. These challenges come from the heteroscedastic nature of count-based compositional data and the potentially low sample size. We identified outliers in all single-cell RNA sequencing datasets we have reanalyzed.

Assessing the adequacy of a statistical model for the query dataset is crucial in real-world analyses. Our method offers a convenient functionality for posterior-predictive checks. Being able to generate data from a fitted model, sccomp offers a data simulation framework that reflects the properties of any target dataset while also allowing arbitrary simulation designs. Data simulation is possible using the sum-constrained Beta-binomial, Dirichlet-multinomial and logit-linear-multinomial distributions.

Our realistic benchmarks show that sccomp confers an up to twofold incremental performance gain compared to previous methods. Our reanalysis of public data demonstrates the practical application and efficacy of sccomp, which identified differential variability and compositional associations. We show that some of the differential composition associations proposed by the respective studies might be false-negative due to the presence of outliers. For the breast cancer dataset introduced by Wu et al. (3), we identify differential constraints for triple-negative, ER+ and HER2+ subtypes.

This study introduces several innovations in cellular omics compositional analysis, such as differential variability analysis, a log-linear mean–variability relation, probabilistic outlier identification, and cross-study information transfer. Also, this study challenges established methodologies and provides a robust and flexible tool for the single-cell and microbiome scientific community. Being a statistical model that fits count data compositionality and group-wise variability while allowing the exclusion of outliers, we anticipate its adoption in other scientific fields. sccomp is available as an R package via Bioconductor and GitHub.

## Methods

### Statistical Model.

sccomp’s regression model is based on Beta-binomial distributions that are sum-constrained and independent. The Beta distribution is continuous and goes from 0 to 1. A binomial distribution models the number of successful outcomes in a set number of trials with an equal probability of success. A Beta-binomial distribution is created by using a Beta distribution for the success probability in a binomial distribution. Unconstrained Beta-binomial distributions can model proportionality but not data compositionality, where proportions must add up to 1. This requirement causes small negative correlations among the proportions of elements across groups, like a multinomial distribution. We impose this negative correlation by requiring the expected values of group proportions (i.e., means of the Beta distributions) to sum to 1.

We introduce here the common notation used in the mathematical formulation of the model. G is the number of groups, S is the number of samples, n_s_ is the total number of cells probed for sample s, k_g,s_ is the number of cells in sample s belonging to a group g. For clarity, we introduce our model in four steps. First, we describe the single-mean model; second, we describe the single-mean model with a log-linear constraint between variabilities and means; third, we introduce a two-mean model; fourth, we describe the linear model generalization used in sccomp.

The Beta-binomial distribution is commonly defined using the (latent) shape parameters ɑ and β (Eq. **1**) from the Beta distribution. Here and elsewhere, B(ɑ, β) denotes the classical Beta function with argument ɑ and β (i.e., $B\left(\alpha ,\;\beta \right)=\int_{0}^{1}t^{\alpha -1}{\left(1-t\right)}^{\beta -1}dt$ ). Here, we use the mean and concentration (the reciprocal of variability, the term used in the *Results* section as more intuitive) parameterization (π,σ) with *π*_g_ representing the mean and σ_g_ representing the concentration (1/σ_g_ representing the variability) parameter of cell group g, this being the sum of the corresponding α and β. This parameterization is convenient for our linear modeling. The mean is the average value of the underlying Beta distribution, while the concentration captures how concentrated the underlying Beta distribution is around its mean. The equivalence of the standard (ɑ, β) and the alternative (π,σ) parameterization is shown in Eqs. **1**–**3**.

[1] BetaBinomial∗kg,s|ns,αg,βg=nskg,sBkg,s+αg,ns-kg,s+βgBαg,βg

[2] $$\alpha_{g}=\pi_{g}\sigma_{g};\beta_{g}=\left(1-\pi_{g}\right)\sigma_{g}\;\mathrm{for}\;0<\pi_{g}<1\;\mathrm{and}\;\sigma_{g}>0$$

[3] BetaBinomialkg,s|ns,πg,σg=nskg,sBkg,s+πgσg,ns-kg,s+1-πgσgBπgσg,1-πgσg

Step 1: Single-mean model. The parameters of the single-mean model are elements ***π*** = (*π*_g_) ∈ S_G+1_ (simplex) of the sum-to-one-constrained vector of size G and a vector **σ** = (σ_g_) ∈ R_+_^G^ of concentrations. The data are a G×S matrix **K** = (k_g,s_) of counts, and a vector **n** = (n_s_) of length S is the sum of **k_s_** ( $n_{s}=\sum_{g=1}^{G}k_{g,s}$ ). The joint probability mass function is defined by two observed quantities, **K** and **n**, depending on the parameters ***π*** and **σ**, see (Eqs. **4**–**7**). Statement 5 includes the sum constraint that induces the weak negative correlation of proportions characteristic of compositional data. The underlying assumption of this model is that the counts k_g,s_ from the total counts n_s_ are mutually independent Beta-binomially distributed random variables with the alternative parameters given.

[4] $$P\left(\pi ,\sigma \right)\prod_{s=1}^{S}\prod_{g=1}^{G}P\left(k_{g,s}n_{s},\pi_{g},\sigma_{g}\right)$$

[5] $$\begin{array}{c} {\text{k}}_{\text{g},s}∼\text{BetaBinomial}\;(n_{\text{s}},\pi_{\text{g}},\sigma_{\text{g}})\;\text{for}\\ \;\sum_{g=1}^{G}\pi_{g}=1\;\text{and}\;\sigma_{\text{g}}>0,\;\text{g}=1,\cdots ,\text{G} \end{array}$$

[6] $$\begin{array}{c} \pi =\mathrm{InverseMultinomialLogistic}\;\left(\mu \right)=\frac{e^{\mu }}{\sum_{g=1}^{G}e^{\mu_{g}}}\;\mathrm{for}\;\sum_{g=1}^{G}\;\mu_{g}=0 \end{array}$$

[7] $$\sigma_{g}=e^{\omega_{g}}$$

Step 2: Single-mean model with a (Log) linear relation between concentrations and means. For this model, we transform the parameters ***π*** and **σ** to **μ** and **ω** (see Eq. **6** and below; −**ω** representing the log variability, the term used in the *Results* section as more intuitive). The parameters ***π*** and **σ** are suitable for an unconstrained single-mean model. Still, to permit a (log) linear relationship between our mean and concentration (the inverse of variability) parameters and the extension to more general linear models, we must use a different but equivalent set of parameters appropriate for linear subspaces of R^G^. The inverse-logit-multinomial (also known as *softma*x) function (Eq. **6**) takes a vector **μ** ∈ R^G^ and converts it into a vector of G proportions that sum to 1, the components being proportional to the exponentials of the corresponding components of **μ**. However, this mapping is many-to-one. If inverse-logit-multinomial (**μ**) = ***π***, then also inverse-logit-multinomial (**μ** + c**1**_M_) = ***π***, where c is any real constant and **1**_M_ is the G-vector of 1s. To make it one-to-one and permit invertibility on its range, we need to restrict its domain. Write ℒ_0,G_ for the linear subspace of R^G^ consisting of all **μ** = (μ_g_) such that $\sum_{g=1}^{G}\;\mu_{g}\;=\;0$ . We will see that for every ***π*** ∈ S_G+1_, there is a unique **μ** ∈ ℒ_0,G_ such that inverse-logit-multinomial (**μ**) = ***π***. We call the **µ** the *logit-multinomial proportion mean* parameters or just *mean* parameters when no confusion is likely. Letting GM denote the geometric mean, we write GM (***π***) = $\sqrt[{G}]{\pi_{1}\pi_{2}\ldots \pi_{G}}$ . Then μ = (μ_g_) where μ _g_ = log (π_*g*_/GM (*π*)) is readily checked to satisfy our requirements, i.e., **μ** ∈ ℒ_0,G_ and *softma*x(**µ**) = inverse-logit-multinomial (**μ**) = ***π***. This function of ***π*** is known as its center (ed) log-ratio (CLR). From (**7**), we see that ω_g_ = log (σ_g_), so our new parameter space is ℒ_0,G_ x R^G^. This process, also known as stick-breaking, underlies the Dirichlet process (40, 41).

[8] $$E\left(\omega_{g}\right)=\lambda_{0}+\lambda_{1}\mu_{g}$$

[9] $$\begin{array}{c} P\left(\mu ,{\text{λ}}_{0},\text{λ}\right)\prod_{g=1}^{G}P\left({\text{ω}}_{g}|\mu_{g},{\text{λ}}_{0},\text{λ},\phi \right)\prod_{s=1}^{S}\prod_{g=1}^{G}P\left(k_{g,s}|n_{s},\mu_{g},\omega_{g}\text{​}\right) \end{array}$$

[10] $$\begin{array}{c} k_{g,s}∼\mathrm{BetaBinomial}\left(n_{s},\mathrm{InverseMultinomialLogistic}{\left(\mu \right)}_{g},e_{g}^{\omega }\right)\\ \;\mathrm{for}\sum_{g=1}^{G}\;\mu_{g}=0 \end{array}$$

Given **µ**, the parameter **ω** will be given a normal prior distribution. The linear relation between **μ** and **ω** which underlies our development is shown in Eq. **8**. where λ_0_ and λ_1_ are scalars. The likelihood and priors for the single-mean model with log-linear concentration-mean relation are represented by formulae **8** and **9**. The complete parameter set is now **μ** ∈ ℒ_0_ ⊂ R^G^, ω ∈ R^G^, λ_0_ ∈ R, λ_1_ ∈ R, and the SD *Φ* ∈ R^+^ going with the normal conditional distribution of the ω_g_s given the µ_g_s; see (**11**). The dataset is unchanged from the original single-mean model. Before generalizing this model, we introduce and use the matrix *M =* (*µ_g,s_*) of mean parameters, where *µ_g,s_* is the mean parameter for sample *s* and group *g*. The single-mean model is characterized by M having all its columns identical.

[11] $$\omega_{g}∼\mathrm{Normal}\left(\lambda_{0}+\lambda_{1}\mu_{g},\phi \right)$$

Step 3: Two-mean model. We now introduce the two-mean model. In this case, the matrix *M* = (*µ_g,s_*) has two potentially distinct types of columns, one for each of two sets of samples. For simplicity, we will call these the control and treated samples and introduce the *2*×*S* matrix X, whose two rows are the indicator vectors (i.e., vectors of zeros and ones) of the control and treated samples, respectively. If we now define a *G*×*2* matrix *Γ* whose columns are any two mean parameter vectors, say ***μ_c_*** ∈ ℒ_0_, ***μ_t_*** ∈ ℒ_0_, then our two-mean model has matrix *M* = *ΓX*.

Step 4: Arbitrary linear model. The approach of the previous paragraph can easily be generalized to arbitrary linear models. For this generalization, we replace the *2*×*S* design matrix *X* above with an arbitrary *C*×*S* design matrix *X*, where *C* is the number of covariates associated with the samples (including one for an intercept if that is appropriate), and the *G*×*2* matrix *Γ* above becomes a general *G*×*C* matrix whose C columns are all elements of ℒ_0_. As before, *M* = *ΓX*.

[12] $$\begin{array}{c} P\left(\text{Γ},{\text{λ}}_{0},\text{λ},\phi \right)\prod_{g=1}^{G}P\left(\omega_{g}|{\text{γ}}_{g,1},{\text{λ}}_{0},{\text{λ}}_{1},\phi \right)\prod_{s=1}^{S}\prod_{g=1}^{G}P\left(k_{g,s}|n_{s},\mu_{g,s},X_{s},\omega_{g}\text{​}\right) \end{array}$$

[13] $$\begin{array}{c} k_{g,s}∼\mathrm{BetaBinomial}\left(n_{s},\mathrm{InverseMultinomialLogistic}{\left(\mu_{s}\right)}_{g},e_{g}^{\omega }\;\right) \end{array}$$

[14] $$\omega_{g}∼\mathrm{Normal}\left(\lambda_{0}+\lambda_{1}\gamma_{g,1},\phi \right)$$

[15] $$\gamma_{g,c}∼\mathrm{Normal}\left(0,5\;\right)$$

[16] $$\lambda_{0},\lambda_{1},∼\mathrm{Normal}\left(0,5\;\right)$$

[17] $$\phi ∼\mathrm{Gamma}\left(20,40\;\right)$$

We now define the full hierarchical linear model based on the sum-constrained Beta-binomial distribution. This model is defined through the *G×C* parameter matrix *Γ;* **φ** of length *G*; *φ*, the scalars λ_0_ and λ_1_; and the dataset includes the *G×S* matrix ***K*** of counts, the *S×1* vector **n** of totals, and the *C×S* design matrix *X*. The prior normal distributions are parameterized by their means and SDs. *X_s_* denotes the design vector (sth column) for sample s, and γ_g_ indicates the coefficient vector (gth row) of *Γ* for cell-group g. Since *M = ΓX*, we must have *µ_g,s_ = γ_g_X_s_*.

#### Inference.

This set of sampling statements and the data (Formulae **12**–**17**) are provided to Stan (23) to sample from a joint posterior distribution of the model parameters. Stan uses a dynamic Hamiltonian Monte Carlo sampling algorithm, a variation on the Markov chain Monte Carlo sampling method. By default, four Markov chains are run. The number of burn-in iterations is 300 for each chain, and the number of sampling iterations is 500 per chain, giving a base of 50 draws for the 2.5% and 97.5% quantiles.

The probability of the null hypothesis (i.e., no effect across conditions) for each group is obtained by estimating the posterior probability of γ_g,c_ (or any combination thereof if contrasts are specified) being larger or smaller than a fold-change threshold (0.2 by default). The false-discovery rate is obtained by sorting in ascending order the probability of the null hypothesis (for any coefficient) and calculating the cumulative average as described by Stephens (24). The existence of an association between cell-group proportions and a factor including three or more categories (analogously to one-way ANOVA) is estimated by comparing the predictive errors between the model with a three-category design and a model with a one-mean design (intercept only). This comparison is achieved through leave-one-out cross-validation (R package loo (42)) and calculating approximate SEs for estimated predictive errors.

### Differential Variability Analyses.

The data variability is modeled by default with one concentration (inverse of variability) parameter ω_g_ per group (variability independent of covariates). However, using a variability design matrix, the user can provide a more general variability model. For example, the concentration can be estimated conditional on a factor of interest to perform differential variability analyses. We now introduce a two-group differential variability model. The following notation is the same as in the paragraph “Step 3, Two-mean model.” of the *Methods* subsection “*Statistical model*”. As ω_g_ is the log concentration for the cell-group g, we introduce ω_g,i_ as the concentration for cell-group g and condition i. In this model, we increase the dimensionality of **ω** from G to 2G, where each ω_g,1_ and ω_g,2_ represents the concentration of group g for two conditions (e.g., treatment and control). The expected value of ω for a two-group differential model and the prior distribution is described in Eqs. **18** and **19**).

[18] $$E\left(\omega_{g,i}\right)=\lambda_{0}+\lambda_{1}\mu_{g,i}$$

[19] $$\omega_{g,i}∼\mathrm{Normal}\left(\lambda_{0}+\lambda_{1}\mu_{g,i},\phi \;\right)$$

Since group proportion means and variabilities are associated (see *Proportion means and variabilities are log-linearly correlated in cell-omic data*), differences in composition and variability will be associated. To test the biological effects that lead to differential variability that is not explained by differences in composition, we need to subtract the contribution of differential composition from the apparent differential variability. We compute the adjusted differential variability (independent of differential composition) using Formula (20). The left side of the formula represents the (apparent) difference between variabilities, and the right side represents the contribution of differential composition.

[20] $$\omega_{g,2}-\omega_{g,1}-\lambda_{1}\left(\mu_{g,2}-\mu_{g,1}\right)$$

Using the Wu et al. dataset, we show that without adjustment, the differential variability and composition estimates would appear correlated (*SI Appendix*, Fig. S6). Often, when a cell group is differentially abundant, it seems also to be differentially variable. Again, this difference is the result of the mean–variability association in the first place. Without adjustment, the difference in variability would indirectly inform us about the difference in the composition without learning anything new. We show in Fig. 3, panels *C* and *D* that, using λ_1_ to adjust for the contribution of differential composition, we obtain estimates for differences in variability that are uncorrelated with differences in composition.

These adjusted differential variability estimates are used to carry out a test along the lines of our testing for differential composition (see *Method* section, *Statistical model* subsection).

### Iterative Outlier Detection.

A robust iterative strategy for outlier identification was developed for negative-binomial data from bulk RNA sequencing (43). Outliers can make a model biased and produce distorted estimates. sccomp has a 3-step process to identify and account for outliers. The first two steps locate outliers, while the third estimates associations. In practice, two iterations are enough to identify all outliers across seven datasets. The first step fits the model and calculates 95% credible intervals for each data point from the fitted parameters. Points outside these intervals are labeled as outliers. This method allows for roughly 5% false positives but captures most outliers. In the second step, the model is refitted without the outliers. This produces reliable posterior probability distributions for accurate outlier identification. The posterior predictive distribution is then made by adjusting for observation censoring (43). This adjustment is necessary because eliminating data at the distribution’s tails leads to downward biases for the estimated variance. Credible intervals are calculated from the data distribution, allowing 5% of groups (compared to sample/cell-group pairs of the first step) to include false-positive outliers. This second step achieves a much more accurate outlier detection, for which we can better control the false-positive rate. In the third step, the model is fitted on the data, excluding the outliers to estimate associations between tissue composition and biological conditions. Credible intervals of the model regression coefficients are calculated from the joint posterior distribution. For each credible interval, enough samples are drawn from the posterior distribution to provide support with 100 draws (by default). For example, for a 95% credible interval, a total of 2,000 draws provides 100 draws beyond the 0.025 and 0.975 quantiles.

### Posterior Predictive Check.

sccomp simulates data from a specific fit to observed data using its posterior predictive distribution. The simulated data can then be overlaid onto the observed data to assess the model’s descriptive adequacy. The probabilistic framework Stan (23) is used for data simulation.

### Cross-Dataset Learning Transfer.

By default, our model uses uninformative Gaussian hyperpriors (see the *Statistical model* subsection) on the intercept (λ_0_), slope (λ_1_), and gamma hyperpriors for the SD (*Φ*) of the prior for the concentration parameter **ω**. sccomp offers the possibility of integrating prior knowledge about the mean–variability association from other, previously analyzed datasets by setting informative hyperpriors. We also provide users with hyperpriors for single-cell RNA sequencing, CyTOF, and microbiome data, integrating the information from the 18 analyzed datasets (*SI Appendix*, Table S1). We fit the model and calculate the posterior means and SDs of the three parameters (λ_0_, λ_1_, *Φ*) from these data sources. We set them as the means and SDs of the respective hyperpriors, regarded as mutually independent. We tested the difference in performances across reference datasets simulating data as described in the Benchmark subsection of the *Methods* section but using a sum-constrained Beta-binomial noise model. We compared the default uninformative hyperpriors with an optimal scenario using the same hyperpriors with which the data have been generated [intercept mean = 4.92, intercept SD = 0.12, slope mean = −0.76, slope SD = 0.09, SD (of the mean–variability association) shape (of a gamma distribution) = 37.45, SD rate = 76.65], hyperpriors from a single-cell RNA sequencing dataset (BRCA1 E-MTAB-10043l; intercept mean = 5.82, intercept SD = 0.14, slope mean = −0.89, slope SD = 0.1061705, SD shape = 53, SD rate = 66), and a misleading hyperprior (intercept mean = 10.00, intercept SD = 0.15, slope mean = 1, slope SD = 0.10, SD shape = 37.00, SD rate = 76.00).

### User Interface.

The function for linear modeling takes as input a table of cell counts (Fig. 1*D*) with three columns containing a cell-group identifier, sample identifier, integer count, and the covariates (continuous or discrete). The user can define a linear model with an input R formula, where the first covariate is the factor of interest. Alternatively, sccomp accepts single-cell data containers [Seurat (44), SingleCellExperiment (45), cell metadata, or group size]. In this case, sccomp derives the count data from cell metadata. The output includes the composition and variability estimates, the probability of the effect being larger than 0.2 (by default), false discovery rate statistics, and the Markov chain Monte Carlo convergence measures.

### Study of Mean–Variability Association.

To study the association between the logit-multinomial mean *µ_g_* (where g is one cell group) and log concentration ω_g_ (negative of log variability) across cellular omics technologies, we gathered seven datasets from single-cell RNA sequencing (1–5, 14, 29–32), six from CyTOF (46–51) and six from microbiome (52–57) studies (*SI Appendix*, Table S1). The cell or taxonomic groups were defined in the respective studies. These datasets were analyzed using the design suggested in the respective studies, assuming that the group-wise variability was independent of the covariates. For each dataset, the parameters *µ_g_* and ω_g_ were first estimated using sccomp without imposing any relationship between the two. This setting was obtained using flat, independent priors on *µ_g_* and ω_g_. We calculated the mean, 2.5% and 97.5% quantiles from the posterior distributions of *µ_g_* and ω_g_. We then calculated the correlation between the posterior means of ω_g_ and *µ_g_* using a robust linear model [rlm, MASS (26, 58)]. The residuals of the robust regression (difference between estimated ω_g_ and regression line) were calculated, and their distribution was analyzed.

To assess the shrinkage effect on the concentration ω_g_ of the modeling of its linear relationship with *µ_g_*, we used sccomp, including the prior of the ω_g_ given the *µ_g_*. We calculated the posterior mean and quantiles as we did with the flat independent priors. We then calculated the shrinkage as the ratio of the estimated means of *µ_g_* and ω_g_ for the two runs with or without conditional priors. We model the bimodal distribution along the regression trend (present in single-cell RNA sequencing data) with a mixture regression model having Gaussian distributed errors. The mixture distribution assumes an ordering of the components. The component with a higher intercept (λ_0,high_) is given a 0.9 probability, and the smaller component (λ_0,low_) is given a probability of 0.1. The slope (λ_1_) and the SD (*Φ*) are assumed to be the same for the two components (given our analyses on the single-cell RNA sequencing data with no linear association between the means and variabilities built-in). The implementation of sccomp allows the model of the mean–variability association using a mixture distribution (suggested for single-cell RNA sequencing data).

### Study of the Adequacy of the Model Fitted to Experimental Data.

To assess the adequacy (59) of the sccomp model fit to experimental data, we used the posterior predictive check (27, 28) on seven datasets from single-cell RNA sequencing (1–5, 14, 29–32), six from CyTOF (46–51) and six from microbiome (52–57) (*SI Appendix*, Table S1). For comparison, we performed the inference and analyses using the sum-constrained Beta-binomial and the Dirichlet-multinomial models. We first used sccomp on the cell or taxonomic groups using the designs defined in the respective studies, assuming the concentrations are independent of covariates. We then used the simulation feature of sccomp to replicate those 18 datasets (i.e., posterior predictive distribution). We calculated proportions from the observed and generated counts and compared their distributions (one element being the proportion for one sample-group pair) using linear regression (lm function from R). To assess the presence of any overestimation or underestimation bias conditional on the relative abundance, we compared the slope of the association between observed and generated data with the baseline group abundance (intercept coefficient).

### Data Analysis and Manipulation.

The data analysis was performed in R (60). Data wrangling was done through tidyverse (61). Single-cell data analysis and manipulation were done through Seurat (44) (version 4.0.1), tidyseurat (62) (version 0.3.0), and tidybulk (63) (version 1.6.1). Parallelization was achieved through makeflow (64). Pair plots created with GGally (cran.r-project.org/web/packages/GGally).

## Supplementary Material

Appendix 01 (PDF)

## Data Availability

The method sccomp, and the code used to generate figures and perform analyses have been deposited (https://github.com/stemangiola/sccomp; https://github.com/stemangiola/sccomp/tree/master/dev) (65). Previously published data were used for this work (1–5, 14, 46–50, 52–57, 66).
