# Supplementary material for: sccomp: Robust differential composition and variability analysis for single-cell data
Source: Proc Natl Acad Sci U S A. 2023 Aug 7;120(33):e2203828120. doi: 10.1073/pnas.2203828120 (PMC10438834; doi:10.1073/pnas.2203828120)
Supplement: Supplementary file 1 — Appendix 01 (PDF) [file pnas.2203828120.sapp.pdf]

SI Appendix for sccomp: Robust differential composition and variability analysis for single-cell data

Supplementary Tables

Table S1. Publicly available datasets and studies used for reanalyses. The name column refers to the abbreviations used in the article. Multiple analysis designs tested for a study are represented by multiple row[i]s in the Design column. NA in the Design or Published study results is used if the published study performed no differential composition. None refer to no significant association detected by a study.

Dataset ID	Dataset number	Name	Sample size	Cell group size	Total counts	Design (covariates)	Reference
**Single-cell RNA**							
GSE139829	2	UVM	11	14	59,915	~ metastatic	Durante et al.
SCP1288	4	RCC	9	33	34,326	~ response	Bi et al.
SCP1039	6	BRCA	26	49	100,064	~ is_TNBC	Wu et al.
EGA S00001004481	3	COVID-19	32	24	135,600	~ criticality	Chua et al.
GSE120575	1	SKCM	48	11	16,291	~ response<br>~ treatment	Sade-Feldman et al.
E-MTAB-10043	5	BRCA1	35	52	197,896	~ pseudotime	Bach et al.
**CyTOF**							
FR-FCM-Z2L2	11	cytofRUV	24	20	$8.6x10^{6}$	~ cancer_status + patient + batch	Trussart et al.
CUAnchor	7	Batchadjust	24	20	$12.2x10^{6}$	~ condition + batch	Schuyler et al. .
FR-FCM-ZYL8	8	Bodenmiller	16	8	$1.7x10^{5}$	~ condition + subject	Bodenmiller et al.
FR-FCM-Z2474	10	Cytonorm	40	25	$6.2x10^{6}$	~ condition + batch + subject	Van Gassen et al.
FR-FCM-Z244	12	Hartmann	12	10	$9.1x10^{5}$	~ condition + day + subject	Hartmann et al.
CovP	9	Chevrier	58	12	$1.4x10^{6}$	~ condition + age + sex	Chevrier et al.
**Microbiome**							
Karlsson_2013	14	A-ML	96	156	$379x10^{6}$	~ is_T2D	Karlsson et al.
Gevers_2014	13	B-ML	617	71	$1.5x10^{6}$	~ diagnosis+ body_site + age + sex + race	Gevers et al.
David_2014	17	C-ML	236	113	$13x10^{6}$	~ diet + subjectFood	David et al.
DS_b17cff2c2d	15	B-DB	920	186	$20.8x10^{6}$	~ type + antibiotic + diet + age + subject	Bokulich et al.
DS_53ce4d9a55	16	C-DB	989	196	$23.4x10^{6}$	~ age_group + body_site + subject	Song et al.

DS_bb7b589593	18	D-DB	1006	124	$3.8x10^{6}$	~ type + age + country	Pop et al.

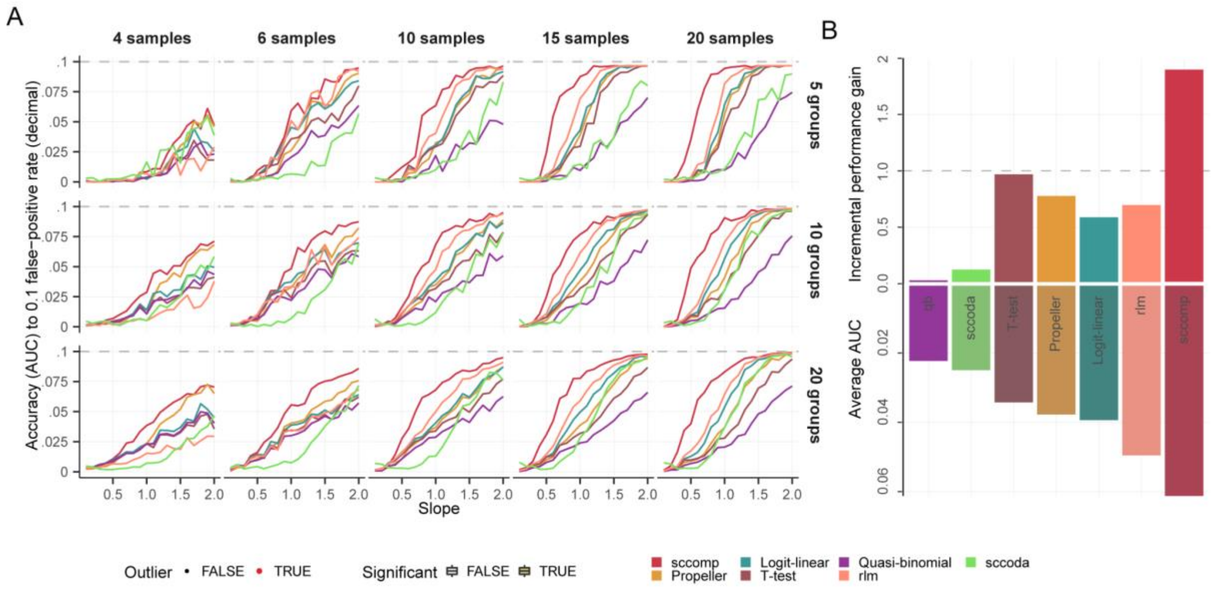

Figure S1. Benchmark on realistic simulated data from the COVID-19COVID19 dataset EGAS00001004481(1) using the sum-constrained Beta-binomial model.

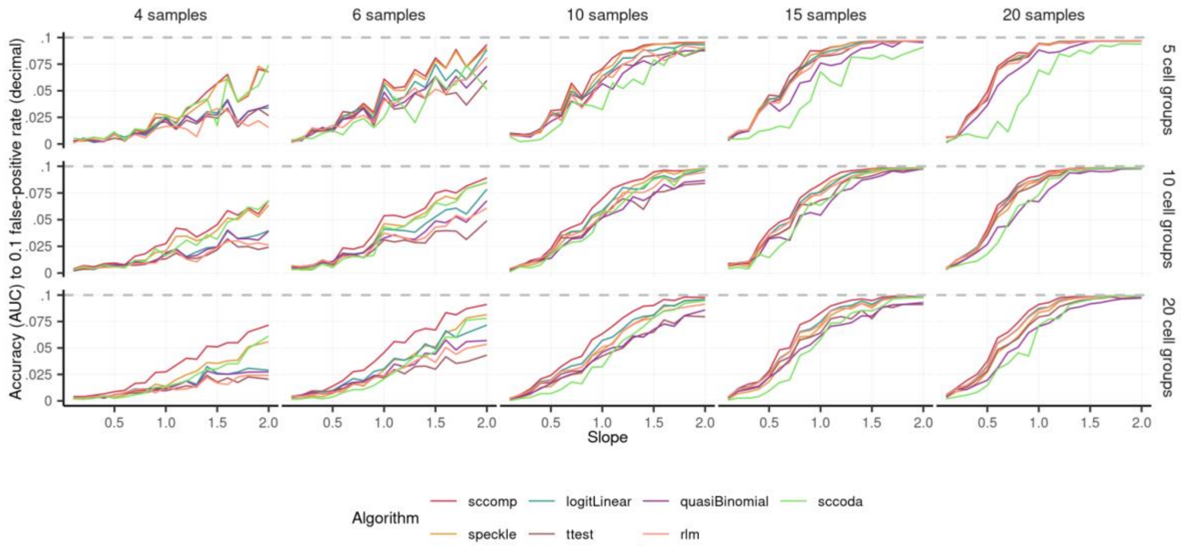

Figure S2. Benchmark on realistic simulated data from the COVID-19COVID19 dataset EGAS00001004481(1) using the outlier-free logit-linear-multinomial model. The comprehensive benchmark across a range of slopes, number of samples and groups. Each performance measure represents an average of 50 areas under the curve (up to the 0.1 false-positive rate) for 50 simulations with the same parameters.

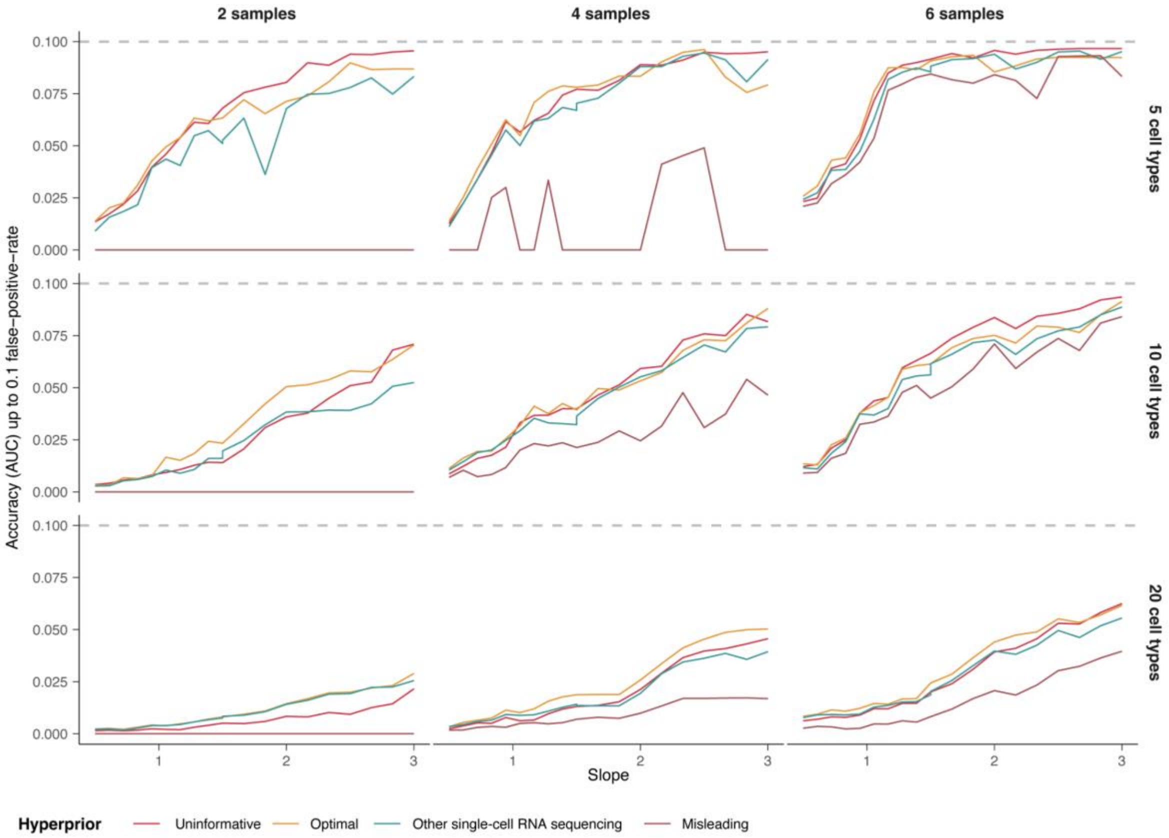

Figure S3. Benchmark for sccomp comparing the use of cross-dataset learning transfer in a low-sample and low-group size setting. Optimal hyperprior is the hyperprior the data was generated with. Other single-cell RNA sequencing refers to the use of a different reference datasets from the same technology of the generating dataset. Misleading refers to a hyperprior that is narrow around wrong value ranges for the proportion mean-variability association.

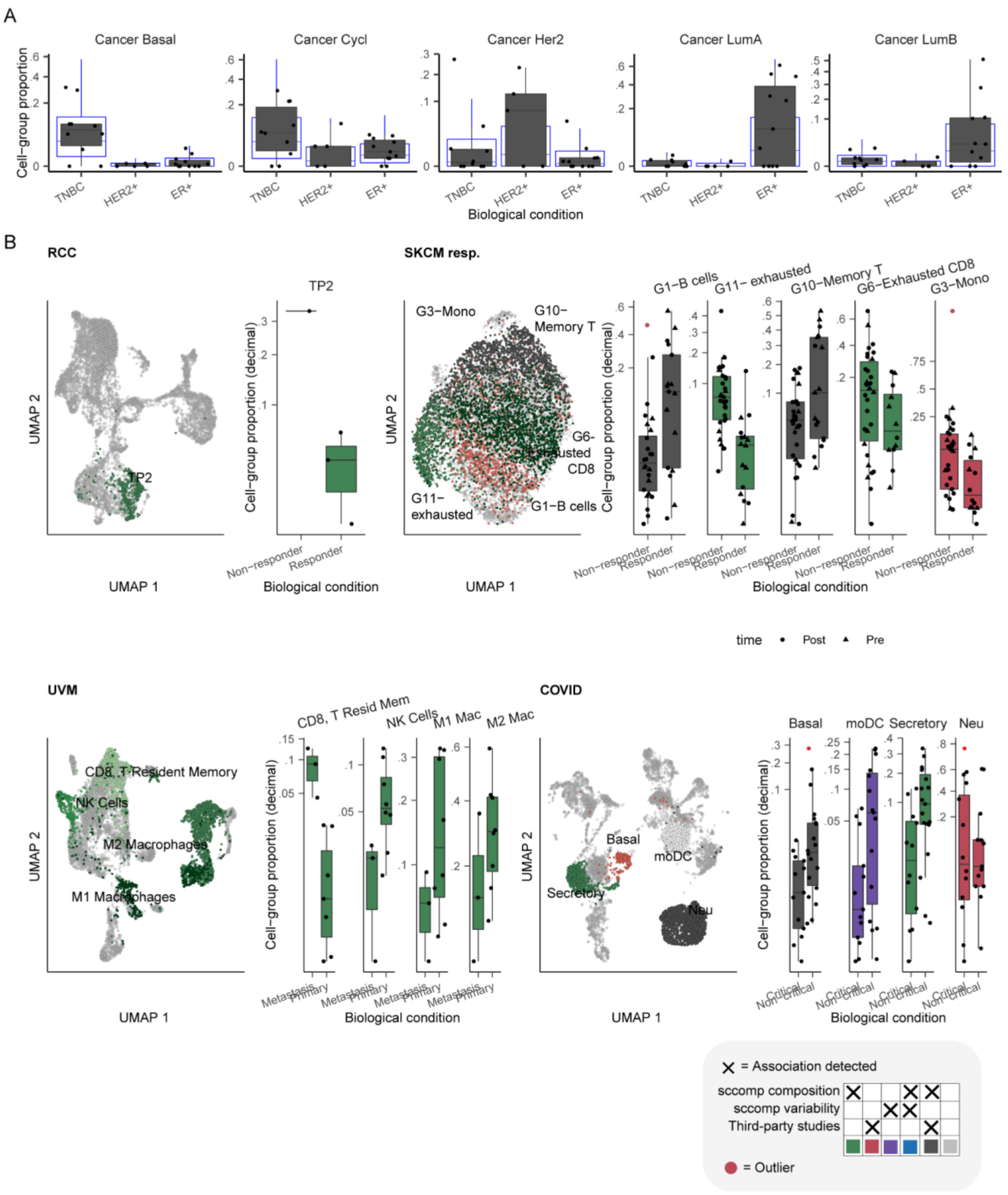

Figure S4. Application of sccomp of publicly available dataset. This figure is a companion to Figure 3. **A:** Proportion distributions of the cell types with the detected associations for cancer populations for the dataset Wu et al.(2). The blue box plots represent the posterior predictive check. **B:** UMAP projection of cells for three breast cancer conditions (subtypes) and boxplots for other datasets (Table S1). Cells are shaded according to the type of finding (e.g. green shades for novel differential composition associations).

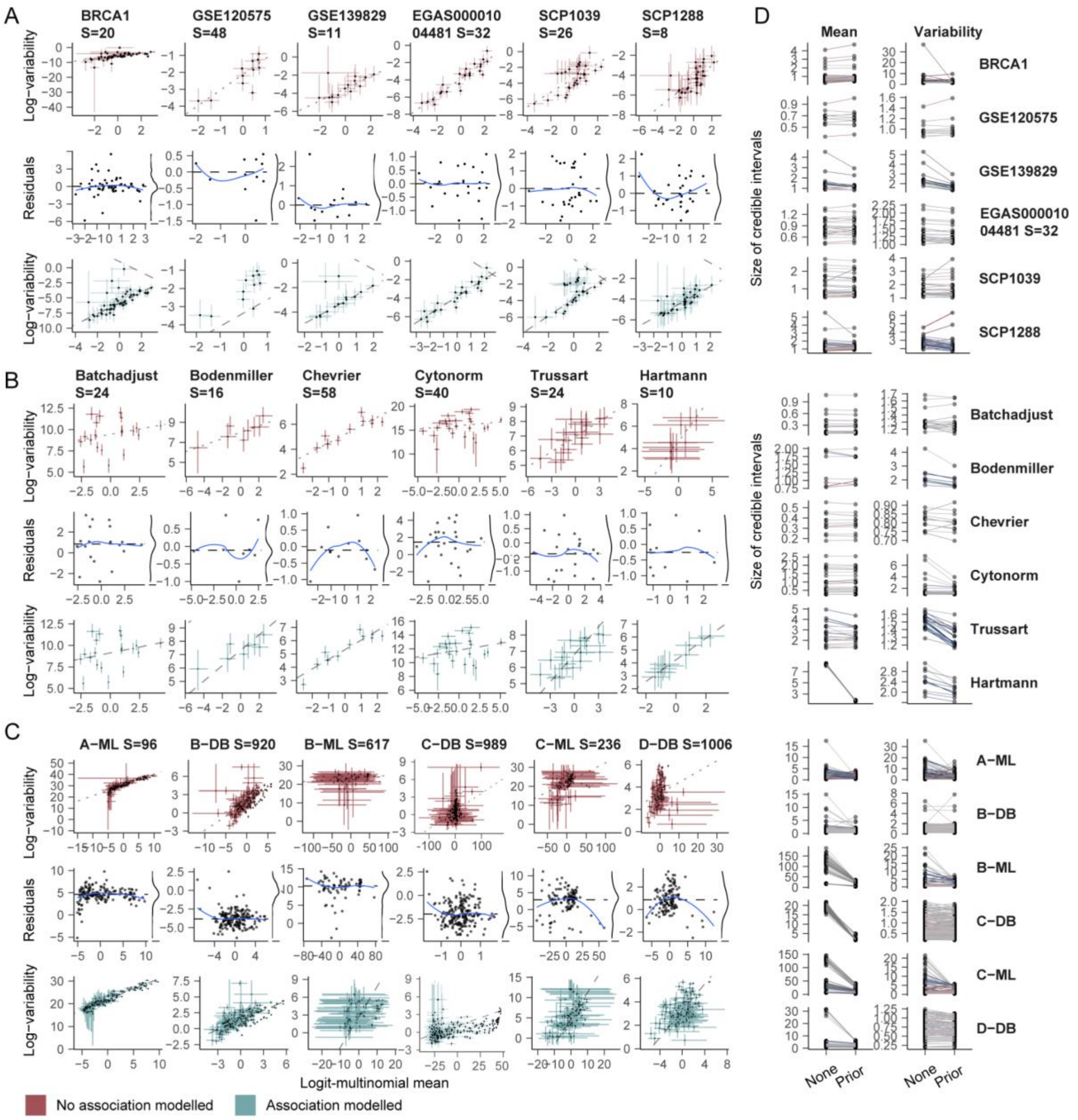

Figure S5. Study of the correlation between the proportion mean and variability from 18 datasets(1–10) (Table S1, see Methods subsection Study of mean-variability association). For panels A, B and C, the points are the posterior means of the parameters. The error bars are the 95% credible intervals. The first row refers to mean and variability estimates association without constraints on their relationship. The dotted line is the line fitted by robust linear modelling (rlm(11)). The second row has rlm residuals vs fitted values with a (blue) lowess smoother superimposed. The third line represents the estimates with the mean-variability association modelled. The dashed lines are the correlation estimated by sccomp. **A:** Mean-variability association for the single-cell RNA sequencing data. For this data type, the bimodal association is modelled (see Methods, Study of mean-variability association) **B:** CyTOF data. The association is modelled as unimodal. **C:** Metagenomics data. The association is modelled as unimodal. **D:** The change in the size of the 95% credible intervals without/with constraints on the mean-variability relationship (None/Prior) for all datasets.

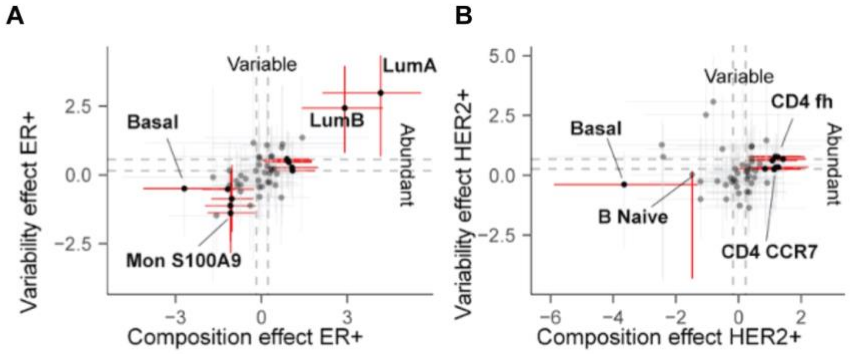

Figure S6. A counterpart of Figure 3 panels C and D (see Methods subsection Differential variability analyses), without adjustment for the mean-variability association. **A:** Estimated difference in composition (x-axis) and variability (y-axis) for the triple-negative versus ER+ comparison, without adjusting the mean-variability association. Error bars are the 95% credible interval. Red error bars represent significant associations. Grey dashed lines represent the minimum difference threshold of 0.2. Significant associations for cancer populations are shown in the supplementary material. **B:** Estimated difference in composition and variability for the triple-negative versus HER2+ comparison without adjusting the mean-variability association.

Supplementary methods

Reanalysis of single-cell RNA sequencing datasets

To assess the ability of sccomp to generate discoveries from publicly available datasets, we applied sccomp to 6 single-cell RNA sequencing datasets(1–6). The cell groups were defined in the respective studies, and the concentration was set to be conditional to the factor of interest (first covariate in the formulae in Table S1). We then compared the significant associations and the presence of outliers identified by sccomp with the findings presented in the respective studies. We estimated the differential composition for all datasets, while we estimated the differential variability only for the datasets with a binary factor of interest and a sample size larger than 10.

For the BRCA (breast cancer) dataset of Wu et al.(2), we went beyond the cell group composition/variability analysis to a higher-level inference. We can think about the mean-variability association as a signature specific to a group of samples (e.g. cancer tissue from patients with a triple-negative breast cancer subtype, TNBC). The intercept of the regression line represents the baseline variability within a tissue across samples. The slope of the regression line represents the uniformity of the variability across cell types. A smaller slope indicates that all groups (cell types) are similarly variable; a larger slope indicates that the larger groups are relatively much more variable than small groups. We can compare these high-level

properties across groups of samples, for example, from different breast cancer conditions (subtypes). This analysis can identify high-level signatures that might underlie biological constraints and processes. For that, we ran sccomp independently for the samples according to the factor of interest "subtype" and compared the posterior distribution of the estimated mean-variability association (λ_1) and the baseline variability (modelled in its negative form as concentration λ_0).

Benchmarking

We base our benchmarking on simulated data with realistic characteristics based on the COVID-19 dataset EGAS00001004481(1). Data was simulated using a logit-linear-multinomial model to ensure fairness across methods. For the simulation, we calculated group proportions across samples and fitted a logit-linear regression model using Stan(12). This model captures the mean-variability association similar to the default sccomp model. We use the posterior distribution to simulate the benchmark datasets, replacing the intercepts and slopes with given ones to establish ground truth. For each simulation, we randomly selected 40% of the groups to be compositionally different between conditions. With the values of the specified parameters, we simulated data from a logit-linear-multinomial model to obtain counts. The total cell count for each sample was set to 1000.

We based the simulation on three variables: the magnitude of the compositional differences, the number of samples (S) per condition (C = 2), and the number of groups (G). The outliers were injected with realistic frequency (10%) and magnitude (from 2 to 10 fold increase or decrease), observed in our data-integration analyses. For each simulation, we produced a receiving-operator characteristic (ROC) curve ranking groups by their statistics and comparing them with the ground truth of significant/non-significant differentially abundant groups. We calculated the area under the curve (AUC) from the receiving-operator characteristic curves to a 0.1 false-positive rate (grey shade in Figure 2B). For each combination of the simulation parameters, we simulated 50 datasets and averaged across the areas under the curves. For comparative purposes, we performed a benchmarking (as described above) simulating data without outliers and using the sum-constrained Beta-binomial distributions.

We assessed the leap of performance improvement that sccomp provides compared to its next-best in relation to the performance gain of all methods. We call this measure incremental performance gain. An incremental performance gain of one indicates that, compared to the average performance improvement that any method had with its second-best,

a method had the same improvement. This can be thought of as linear incremental performance gain. For each of the 15 simulation settings, we calculated the average area under the ROC curve (AUC) to obtain a unique performance score. From this average, we excluded the slope ranges where the performance of most algorithms approached a plateau to calculate the difference in performance in the most informative simulation regimes. For each simulation setting, we ranked methods based on the performance score. We calculate the gain in performance as the difference in average AUC between each method and their next-best ranked (e.g. first against second, second against third). The fold gain in performance was calculated as the ratio between the gain in performance of each method and the average of all others.

References

1. R. L. Chua, *et al.*, COVID-19 severity correlates with airway epithelium-immune cell interactions identified by single-cell analysis. *Nat. Biotechnol.* **38**, 970–979 (2020).

2. S. Z. Wu, *et al.*, A single-cell and spatially resolved atlas of human breast cancers. *Nat. Genet.* **53**, 1334–1347 (2021).

3. M. A. Durante, *et al.*, Single-cell analysis reveals new evolutionary complexity in uveal melanoma. *Nat. Commun.* **11**, 496 (2020).

4. K. Bi, *et al.*, Tumor and immune reprogramming during immunotherapy in advanced renal cell carcinoma. *Cancer Cell* **39**, 649-661.e5 (2021).

5. M. Sade-Feldman, *et al.*, Defining T Cell States Associated with Response to Checkpoint Immunotherapy in Melanoma. *Cell* **175**, 998-1013.e20 (2018).

6. K. Bach, *et al.*, Time-resolved single-cell analysis of Brca1 associated mammary tumourigenesis reveals aberrant differentiation of luminal progenitors. *Nat. Commun.* **12**, 1502 (2021).

7. S. Freytag, L. Tian, I. Lönnstedt, M. Ng, M. Bahlo, Comparison of clustering tools in R for medium-sized 10x Genomics single-cell RNA-sequencing data. *F1000Research* **7**, 1297 (2018).

8. J. Ding, *et al.*, Systematic comparison of single-cell and single-nucleus RNA-sequencing methods. *Nat. Biotechnol.* **38**, 737–746 (2020).

9. T. T. Karagiannis, *et al.*, Single cell transcriptomics reveals opioid usage evokes widespread suppression of antiviral gene program. *Nat. Commun.* **11**, 2611 (2020).

10. Y. Cai, *et al.*, Single-cell transcriptomics of blood reveals a natural killer cell subset depletion in tuberculosis. *EBioMedicine* **53**, 102686 (2020).

11. C. Jennison, F. R. Hampel, E. M. Ronchetti, P. J. Rousseeuw, W. A. Stahel, Robust Statistics: The Approach Based on Influence Functions. *Journal of the Royal Statistical Society. Series A (General)* **150**, 281 (1987).

12. B. Carpenter, *et al.*, Stan: A Probabilistic Programming Language. *Journal of Statistical Software* **76** (2017).

[i]
